# Loss of the type VII secretion ATPase EssC promotes biofilm formation of *Staphylococcusaureus* under acidic stress

**DOI:** 10.1016/j.bioflm.2026.100376

**Published:** 2026-06-23

**Authors:** Yang Zhang, Zihui Wang, Lan Wei, Deyu Wang, Di Qu, Youhua Xie, Yang Wu

**Affiliations:** Key Laboratory of Medical Molecular Virology (MOE/NHC/CAMS), School of Basic Medical Sciences, Shanghai Medical College, Fudan University, Shanghai, China

**Keywords:** *Staphylococcus aureus*, Type VII secretion system, EssC, Acidic stress, Biofilm

## Abstract

*Staphylococcus aureus* adapts to hostile host-associated niches by dynamically switching between planktonic growth and biofilm lifestyles. Acidic environments, such as the skin surface and intracellular compartments, impose substantial stress on bacterial survival; however, the contribution of the type VII secretion system (T7SS) to biofilm adaptation under acidic conditions remains poorly understood. Here, we investigated the role of the T7SS ATPase EssC in regulating *S. aureus* biofilm formation under acidic stress. Using an *essC* deletion mutant in the USA300 background, we demonstrate that loss of EssC markedly enhances biofilm biomass and thickness at pH 5.0, despite reducing bacterial viability within mature biofilms. Mechanistically, *essC* deletion reprograms multiple stages of biofilm development, including enhanced initial adhesion mediated by upregulated fibronectin-binding proteins (FnBPA and FnBPB), increased intercellular aggregation driven by elevated polysaccharide intercellular adhesin (PIA) production, and biofilm stabilization through augmented autolysis-dependent extracellular DNA release. These phenotypic changes are accompanied by coordinated transcriptional remodeling, characterized by downregulation of the biofilm repressor *agr* and activation of the *arlS–icaA* and *sigB–icaA* regulatory axis. Collectively, our findings uncover an unrecognized link between the T7SS core component EssC and biofilm regulation under acidic stress, highlighting EssC as a potential modulator of *S. aureus* survival strategies in hostile host microenvironments.

## 1 Introduction

*Staphylococcus aureus* is a versatile human pathogen capable of colonizing and infecting diverse host niches that differ markedly in physicochemical properties [1,2], including pH [[3], [4], [5]], osmolarity [6], temperature [7,8], and nutrient availability [[9], [10], [11], [12]]. Among these, acidic microenvironments (such as the skin surface [3,4], inflamed tissues [5], and intracellular compartments of phagocytic cells [13]) pose a substantial challenge to bacterial growth and persistence. Nevertheless, *S. aureus* successfully adapts to these conditions and causes both acute and chronic infections [2,14], underscoring the importance of its environmental stress response mechanisms.

One of the most effective adaptive strategies employed by *S. aureus* is the formation of biofilms. Biofilms are structured multicellular communities encased in an extracellular polymeric matrix that confers protection against antimicrobial agents, host immune defenses, and environmental stressors [[15], [16], [17], [18], [19]]. Biofilm development proceeds through sequential stages of initial attachment, intercellular aggregation and maturation, and eventual dispersal [20]. These processes are tightly regulated by a complex network of surface adhesins [[21], [22], [23]], extracellular matrix components [16,17,19,20,24,25], and global regulatory systems [3,20,22,23,[26], [27], [28], [29], [30], [31]]. Despite extensive research on biofilm regulation, how environmental cues such as acidic stress interface with intracellular regulatory pathways to shape biofilm phenotypes remains incompletely understood.

The type VII secretion system (T7SS) is a specialized protein secretion apparatus conserved in Gram-positive bacteria, including *S. aureus* [32,33]. In *S. aureus*, the T7SS gene cluster encodes secreted substrates such as EsxA and EsxB, as well as essential structural components, among which EssC functions as the central ATPase driving substrate secretion. Previous studies have established that EssC is indispensable for T7SS activity and contributes to virulence, immune evasion, and resistance to host-derived antimicrobial factors [[34], [35], [36], [37], [38]]. However, most investigations have focused on the roles of secreted effectors, whereas the contribution of the T7SS core machinery itself-particularly EssC-to environmental adaptation and biofilm regulation has received little attention.

Given the ecological relevance of acidic niches during *S. aureus* colonization and infection, we hypothesized that EssC may influence biofilm formation under acidic stress. In this study, we systematically examined the impact of *essC* deletion on biofilm development, cellular physiology, and regulatory networks in *S. aureus* USA300 under acidic conditions. Our findings reveal that loss of EssC promotes a biofilm-oriented survival strategy through coordinated modulation of adhesion, extracellular matrix production, autolysis, and global regulatory pathways, thereby uncovering a previously unappreciated link between the T7SS and biofilm adaptation.

## 2 Materials and methods

### 2.1 Bacterial strains, plasmids, primers and growth conditions

All bacterial strains and plasmids used in this study are listed in Table 1, and primers are listed in Table 2. *Staphylococcus aureus* USA300 (genome accession number NC_010079), preserved in our laboratory, was used as the wild-type (WT) strain. *S. aureus* NCTC8325 wild-type and Δ*fnbA fnbB* strains were used in the initial attachment assay to clarify the role of FnBPA and FnBPB in the initial adhesion stage of bacteria. *S. aureus* USA300Δ*arlRS* was used in fluorescence reporting system detection to preliminary verify the regulatory relationship between *arlRS* and *essC*. The *icaA* transposon insertion mutant of *S. aureus* USA300 (USA300 Tn::*icaA*) and the *icaC* knockout mutant of *S. epidermidis* 1457 strain (1457Δ*icaC*) as negative control for PIA synthesis.

**Table 1:** Bacterial strains and plasmids used in this study.

Strain or Plasmid	Descriptions
**Strains**	
*S. aureus* USA300 TCH1516	Strain our lab preserved
*S. aureus* USA300 Δ*essC*	An *essC* mutant srain of *S. aureus* USA300
*S. aureus* USA300 Δ*essC* (pLI50-*essC*)	Complementation of *essC* into the Δ*essC*
*S. aureus* USA300 Δ*essC* (pLI50)	Vector control
*S. aureus* NCTC8325	Kindly provided by Dr. Qian Liu in Shanghai Jiao Tong University
*S. aureus* NCTC8325 Δ*fnbA fnbB*	A *fnbA* and *fnbB* double-deletion mutant srain of *S. aureus* NCTC8325 (provided by Dr. Qian Liu)
*S. aureus* USA300 Δ*arlRS*	An *arlRS* mutant srain of *S. aureus* USA300
*S. aureus* USA300 Tn:*icaA*	Strains our lab preserved, used as negative control for PIA synthesis
*S. epidermidis* 1457 *ΔicaC*	
**Plasmids**	
pCM29-P_T7SS_-GFP	The fluorescent reporter plasmid, carrying green fluorescent protein (GFP), was used to detect T7SS promoter activity Cm^R^ Amp^R^
pKOR1	Shuttle vector, temperature sensitive, vector for allelic replacement via lambda recombination and *ccdB* selection Cm^R^ Amp^R^
pLI50	Vector for complementation Amp^R^, Cm^R^

**Table 2:** Primers used in this study.

Primers	Sequence(5'-3')
**Using for *essC* Knockout**	
US-attB1-F	GGGGACAAGTTTGTACAAAAAAGCAGGCTggaacgccatttgaaactaaag
US-KpnI-R	*CGGGGTACC*tgtctttgcctcagtcctatac
DS-KpnI-F	*CGGGGTACC*caatgaattaaataggagggaggtatg
DS-attB2-R	GGGGACCACTTTGTACAAGAAAGCTGGGTcgagaggcaatttactaccacc
**Using for Confirmation of BP**	
BP-F	caacgcaattaatgtgag
BP-R	agtcggttttctaatgtc
**Using for Complementation of *essC***	
pLI50-promoter-XbaI-F	*GCTCTAGA*atgtataagtatacgcgccgg
pLI50-promoter-EcoRI	*GGAATTC*aactagaaacctcctgaatattttaagtt
pLI50-*essC*-EcoRI-F	*GGAATTC*atgcataaattgattataaaatataacaaacaattgaag
pLI50-*essC*-XmaI-R	*TCCCCCCGGG*ctatttaaaccatctaatcttttgataagcttgg
**Using for qRT-PCR of Key Genes Related to Phenotypes**	
*gyrB*-qp-F	gcttgttcaagcaggtcgtg
*gyrB*-qp-R	agctcttcgtctgtccaagc
*agrC*-qp-F	ccctatcattcgcgttgca
*agrC*-qp-R	gacctaaaccacgaccttcacc
*arlS*-qp-F	aaaaccatctcgtcgaatctcaatc
*arlS*-qp-R	tgcgctggcatttggagtg
*sigB*-qp-F	gaaccatctttatcagcttcaatgg
*sigB*-qp-R	gctgatcgattagaagtctcagaag
*icaA*-qp-F	gcctgttataacgaaagtgaaacg
*icaA*-qp-R	ctgctgtattatctgaacttccatc
*atl*-qp-F	cgctaattgagaagtaccgttac
*atl*-qp-R	caaagctgctcaaatgtatggc
*fnbA*-qp-F	gtaccgctcgttgtcctgc
*fnbA*-qp-R	tggaaacgggtacagatgtaacaag
*fnbB*-qp-F	ggttgttaggtttcatgtacgc
*fnbB*-qp-R	ttagagataattggggagtaacagc

The upright capital letters represent the introduction of the *attB* site during PCR amplification. Italicized capital letters represent the introduction of restriction sites during PCR amplification. Lowercase letters represent gene sequences.

All strains were routinely cultured in tryptic soy broth (TSB; Oxoid, Basingstoke, UK) at 37 °C with shaking at 200 rpm unless otherwise indicated, and this condition is also defined as the control group condition (CK). Acidic stress was simulated by adjusting TSB to pH 5.0 using hydrochloric acid (pH 5.0). For biofilm assays, TSB was supplemented with 1% (w/v) glucose. When required, antibiotics were added at the following concentrations: ampicillin (100 μg/mL) and chloramphenicol (10 μg/mL). B2 medium (1% casein hydrolysate, 2.5% yeast extract, 0.5% glucose, 2.5% NaCl, 0.1% K_2_HPO_4_, pH 7.5) was used for recovery after electroporation.

### 2.2 Construction of the mutant strains

The *essC* deletion mutant (Δ*essC*) was constructed using the temperature-sensitive shuttle vector pKOR1 via allelic replacement as previously described. Briefly, upstream and downstream homologous fragments of *essC* were amplified and cloned into pKOR1 to generate pKOR1-Δ*essC* by BP reaction (Gateway™BP Clonase™II enzyme, 16 °C), followed by electroporation into *S. aureus* USA300 (cultured at 30 °C) and selection using temperature shift and antibiotic counter-selection (cultured at 43 °C to integrates the plasmid into the genome, then cultured at 37 °C and induced with anhydrotetracycline to promote the loss of the plasmid). For genetic complementation, the full-length *essC* gene with its native promoter was cloned into the shuttle vector pLI50, yielding pLI50-*essC*, which was introduced into the Δ*essC* mutant. The Δ*essC* strain carrying empty pLI50 served as a vector control.

### 2.3 Biofilm formation assay

Biofilm formation was quantified using a crystal violet staining assay. Overnight cultures were diluted 1:200 into fresh TSB (pH 7.0 or pH 5.0) supplemented with 1% glucose and incubated in 96-well plates (Corning Inc., United States) at 37 °C for 48 h. Wells were gently washed with phosphate-buffered saline (PBS) to remove non-adherent cells, fixed with methanol, stained with 1% crystal violet, and solubilized with 10% acetic acid. Absorbance was measured at 570 nm.

### 2.4 Confocal laser scanning microscopy (CLSM)

Biofilms for microscopic observation were grown in glass-bottom FluoroDishes (WPI, USA) under the same conditions as described above. After 48 h incubation, biofilms were gently washed twice with PBS and stained with SYTO-9 (1 μM) and propidium iodide (PI, 1 μM) (LIVE/DEAD BacLight kit; Invitrogen, USA) to assess cell viability. Polysaccharide intercellular adhesin (PIA) was visualized using wheat germ agglutinin (WGA)–Alexa Fluor 350 conjugate (2.5 μg/mL; Thermo Fisher Scientific, USA). Samples were incubated with dyes for 20 min in the dark at room temperature. Images were acquired using a confocal laser scanning microscope (Leica TCS SP8, Leica Microsystems, Germany). Three-dimensional reconstructions were generated using IMARIS software (Bitplane, Switzerland). Fluorescence intensities were quantified using ImageJ software (NIH, USA), with at least three random fields analyzed per sample.

### 2.5 Initial attachment assay

Initial bacterial attachment was assessed as described previously with minor modifications. Mid-exponential-phase cultures were harvested, washed three times with PBS, and resuspended in TSB (pH 7.0 or pH 5.0) to an OD600 of 0.1. One milliliter of bacterial suspension was added to each well of a six-well polyethylene plate (Nunc, Thermo Fisher Scientific, USA) and incubated at 37 °C for 1 h. Wells were gently washed three times with PBS to remove non-adherent cells. Attached bacteria were visualized under a light microscope (Olympus, Japan) and quantified using ImageJ software from at least three randomly selected fields.

### 2.6 Auto-aggregation assay

Auto-aggregation assays were performed to evaluate intercellular aggregation. Mid-exponential-phase cultures were washed and resuspended in TSB (pH 7.0 or pH 5.0) to an initial OD600 (A_0_) of 1.0. Cell suspensions were incubated statically at 37 °C. At 3 h and 6 h, the upper suspension was carefully collected without disturbing the sedimented cells, and the OD600 (A_t_) was measured. Auto-aggregation percentage was calculated as: Auto-aggregation (%) = (1 − A_t_/A_0_) × 100.

### 2.7 Triton X-100–induced autolysis assay

Bacterial autolysis was assessed using a Triton X-100–induced lysis assay. Overnight cultures were diluted into fresh TSB (pH 7.0 or pH 5.0) containing 1 M NaCl and grown to mid-exponential phase (OD600 = 0.6–0.8). Cells were harvested, washed twice with cold sterile distilled water, and resuspended in 0.05 M Tris-HCl buffer (pH 7.2) containing 0.05% Triton X-100. Cell suspensions were incubated at 30 °C, and OD600 was measured every 30 min for up to 6 h using a spectrophotometer (Bio-Rad, USA). Autolysis was expressed as the percentage decrease in OD600 relative to the initial value.

### 2.8 Extracellular DNA (eDNA) extraction and DNase I treatment

Extracellular DNA was extracted from 48-h biofilms. Biofilms were treated with 0.5 M EDTA at 4 °C for 1 h and scraped into eDNA extraction buffer. Samples were centrifuged, and the supernatant was purified twice using phenol:chloroform:isoamyl alcohol (25:24:1). eDNA was precipitated with sodium acetate and ethanol, resuspended in sterile distilled water, and quantified using a NanoDrop ND-1000 spectrophotometer (Thermo Fisher Scientific, USA). For DNase I treatment, 5 μL DNase I (5 U/μL; Takara, Japan) was added during biofilm formation, and biofilm biomass was quantified as described above.

### 2.9 Cell surface charge determination

Cell surface charge was determined using FITC-labeled poly-L-lysine (FITC-PLL; Sigma-Aldrich, USA). Overnight cultures were washed and resuspended in PBS to an OD600 of 1.0. FITC-PLL was added to a final concentration of 80 μg/mL, and samples were incubated in the dark at room temperature for 10 min. Fluorescence intensity was measured using a Victor X5 multimode plate reader (PerkinElmer, USA) with excitation at 480 nm and emission at 530 nm.

### 2.10 RNA extraction and quantitative real-time PCR

Total RNA was extracted from cultures grown for 16 h under neutral or acidic conditions using the RNeasy Mini Kit (Qiagen, Germany) following the manufacturer's instructions. Residual genomic DNA was removed by on-column DNase I digestion. cDNA was synthesized from 1 μg RNA using the PrimeScript RT reagent kit (Takara, Japan). Quantitative real-time PCR was performed using SYBR Green Premix (Takara, Japan) on a QuantStudio 5 Real-Time PCR System (Applied Biosystems, USA). Gene expression levels were normalized to the housekeeping gene *gyrB*, and relative expression was calculated using the 2^−ΔΔCt^ method.

### 2.11 Construction of the fluorescence reporting system

The wild-type and Δ*arlRS* mutant strains of *Staphylococcus aureus* USA300 carrying the plasmid pCM29- P_T7SS_ -GFP were inoculated into TSB medium containing 10 μg/mL chloramphenicol. The cultures were then diluted 1:200 into control (CK, pH 7.2) and acidic (pH 5.0) TSB media (both supplemented with 10 μg/mL chloramphenicol). After 16 h of incubation, bacterial cells were collected, washed with phosphate-buffered saline (PBS), and adjusted to an OD_600_ of 1.0. The suspensions were then transferred to a black 96-well plate, and fluorescence intensity (FI) was measured using a PerkinElmer Victor X5 microplate reader (Ex/Em = 485/530 nm). The regulatory effect of *arlRS* on the T7SS under acidic conditions was assessed by comparing the fluorescence intensity (FI/OD_600_) between the *arlRS* deletion strain and the wild-type strain, as well as between treated and control groups.

### 2.12 Statistical analysis

All experiments were performed with at least three independent biological replicates. Data were analyzed using GraphPad Prism 7 (GraphPad Software, USA). Statistical significance was determined using unpaired two-tailed Student's t tests or one-way ANOVA followed by Bonferroni's post hoc test, as appropriate. A P value < 0.05 was considered statistically significant.

## 3 Results

### 3.1 Deletion of essC markedly enhances biofilm formation of *S. aureus* under acidic conditions

**Fig. 1:**
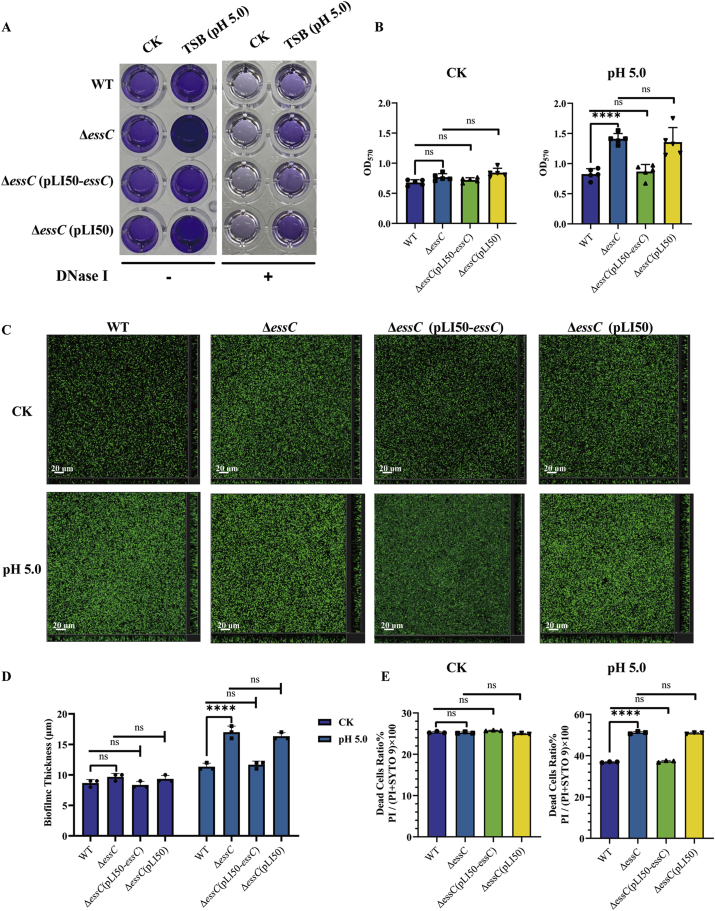

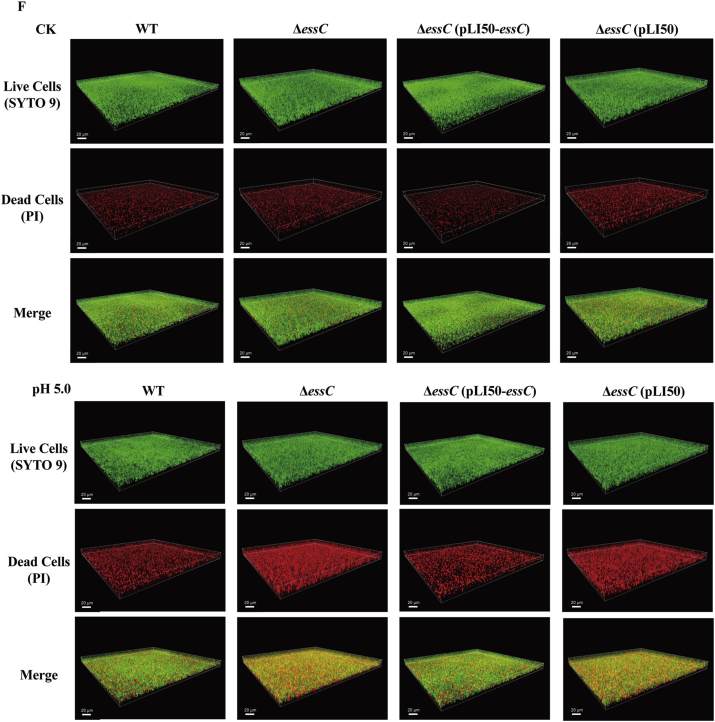
Deletion of *essC* markedly enhances biofilm formation of *S. aureus* under acidic conditions (**A**) Crystal violet staining of biofilms formed by the *S. aureus* USA300 WT and *essC* mutants in TSB (CK) and TSB (pH 5.0) at 37 °C for 48 h, without (left) or with (right) DNase I treatment. After crystal violet staining, the biofilms were dissolved in 10% acetic acid. DNase I was added to the wells (25 U/well) in DNase I treated groups. (**B**) Quantitative analysis of biofilm formation of *S. aureus* USA300 WT and *essC* mutants after crystal violet staining by measuring the OD570. Statistical significance was determined by one-way ANOVA (n = 5; ∗∗∗∗: *P* < 0.0001; ns: No difference) (**C**) *S. aureus* USA300 WT and *essC* mutants biofilms under the control and acidic environment were staining with SYTO9and observed by confocal laser scanning microscopy (CLSM). (**D**) The thickness of biofilms of *S. aureus* USA300 WT and *essC* mutant strains under the control and acidic environment was analyzed using Leica Application Suite 1.0 software. Statistical significance was determined by one-way ANOVA (n = 3; ∗∗∗∗: *P* < 0.0001; ns: No difference). **(E)** Quantification of dead cells ratio within the biofilms of *S. aureus* USA300 WT and *essC* mutants, measured by the formula: Dead Cells Ratio (%) = PI/(PI + SYTO-9) ×100. **(F)** Representative fluorescence images of three-dimensional architecture of 48-h biofilms formed by *S. aureus* USA300 WT and *essC* mutant strains under the control and acidic environment by Live/Dead staining. (For interpretation of the references to colour in this figure legend, the reader is referred to the Web version of this article.)

To determine whether EssC contributes to biofilm formation under acidic stress, *S. aureus* USA300 WT, the Δ*essC* mutant, the complemented strain Δ*essC*(pLI50-*essC*), and the vector control were cultured in TSB adjusted to pH 7.0 or pH 5.0 and supplemented with 1% glucose for 48 h. Biofilm biomass was quantified using crystal violet staining. Under neutral conditions, no significant differences in biofilm formation were observed among the strains. In contrast, exposure to acidic conditions (pH 5.0) resulted in a pronounced increase in biofilm biomass in the Δ*essC* mutant compared with the WT strain, with approximately a 1.5-fold increase in OD570 values (OD_570 WT:Δ*essC*_ = 0.768 ± 0.058: 1.411 ± 0.089, *P* < 0.05). Complementation of *essC* largely restored biofilm formation to WT levels, whereas the vector control retained the hyper-biofilm phenotype, confirming that the observed effect was specifically attributable to loss of EssC (Fig. 1A and B).

To further characterize biofilm architecture, confocal laser scanning microscopy (CLSM) was employed to visualize mature biofilms. Three-dimensional reconstructions revealed that biofilms formed by the Δ*essC* mutant under acidic conditions were substantially thicker and more densely structured than those of the WT strain (Fig. 1C and D). Quantitative analysis showed that biofilm thickness increased from approximately 11 μm in the WT strain to ∼16 μm in the Δ*essC* mutant at pH 5.0, whereas complementation again reversed this phenotype (Table 3). These results indicate that EssC negatively regulates biofilm accumulation and maturation specifically under acidic stress.

**Table 3:** Biofilm thickness.

Srains	Thickness (μm)	
	CK	pH 5.0
**WT**	8.83 ± 0.29	11.83 ± 0.76
**Δ*essC***	9.50 ± 0.00	16.00 ± 0.5
**Δ*essC*(pLI50-*essC*)**	8.83 ± 0.29	12.33 ± 0.29
**Δ*essC*(pLI50)**	9.33 ± 0.29	15.67 ± 0.29

### 3.2 Loss of EssC reduces bacterial viability within mature biofilms

Although deletion of *essC* promoted biofilm accumulation under acidic conditions, the physiological state of bacteria within these biofilms was further assessed. Mature biofilms were stained with SYTO-9 and propidium iodide to distinguish viable from non-viable cells. CLSM imaging revealed a marked increase in propidium iodide–positive cells within Δ*essC* mutant biofilms formed at pH 5.0, indicating a higher proportion of dead or membrane-compromised cells (Fig. 1E and F).

Quantification of fluorescence intensities demonstrated that more than half of the bacterial population within Δ*essC* mutant biofilms exhibited reduced viability under acidic conditions, whereas WT biofilms retained a significantly higher proportion of viable cells (Fig. 1E). These findings suggest that loss of EssC imposes a growth or survival disadvantage under acidic stress, which is accompanied by a compensatory shift toward enhanced biofilm formation.

### 3.3 Deletion of essC promotes PIA production and intercellular aggregation via transcriptional remodeling

**Fig. 2:**
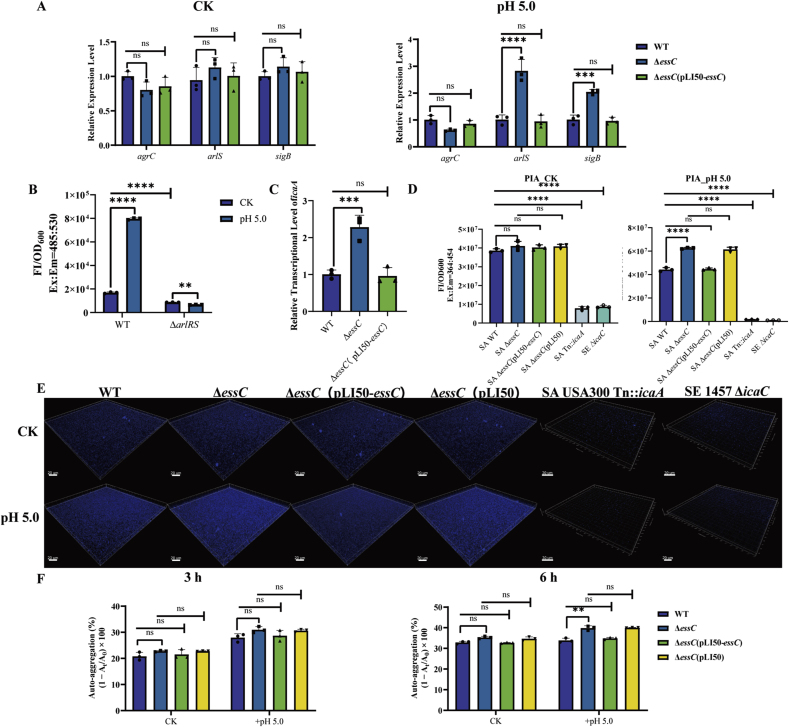
Deletion of *essC* promotes PIA production and intercellular aggregation via transcriptional remodeling (**A**) Detection of various regulatory genes under the control and acidic environment by qRT-PCR, including *agrC, arlS* and *sigB,* related both to the *essC* and biofilm formation. No obvious change observed in the control group, while the expression level of *agrC* slightly decreased, while *arlS* and *sigB* strongly upregulated in acidic environment. Statistical significance was determined by one-way ANOVA (n = 3; ∗∗∗: *P* < 0.0005; ∗∗∗∗: *P* < 0.0001; ns: No difference). (**B**) The changes of the activity of the T7SS promoter in Δ*arlRS* (Promoter-Fluorescence reporting system). After *arlRS* knockout, the phenomenon of acidic activation of T7SS transcription disappeared, and the activity of the T7SS promoter in Δ*arlRS* knockout strains decreased compared with the control group. Statistical significance was determined by Unpaired *t*-test (n = 3; ∗∗∗: *P* < 0.0005). **(C)** In the Δ*essC* knockout strain, the expression of the *icaA* (which is involved in PIA biosynthesis) were significantly upregulated. Statistical significance was determined by one-way ANOVA (n = 3; ∗∗∗: *P* < 0.0005; ns: No difference). **(D)** Determination of PIA content in the *icaA* transposon insertion mutant of *S. aureus* USA300 (USA300 Tn::*icaA*), the *icaC* knockout mutant of *S. epidermidis* 1457 strain (1457Δ*icaC*) and *S. aureus* USA300 WT and *essC* mutants biofilms in control and acidic-treated conditions, measured by the fluorescent intensity after WGA probe staining. (Fluorescence quantification). Statistical significance was determined by one-way ANOVA (n = 3; ∗∗∗∗: *P* < 0.0001; ns: No difference). **(E)** Representative fluorescence images of three-dimensional architecture of 48-h biofilms formed by *S. aureus* USA300 WT and *essC* mutant strains and *S. aureus* USA300 Tn::*icaA* and *S. epidermidis* 1457Δ*icaC* under the control and acidic environment by WGA probe staining. **(F)** The percentage of aggregation of *S. aureus* USA300 WT and *essC* mutants under the control and acidic conditions for 3 h and 6 h, calculated by the formula: Auto aggregation (%) = (1−A_T_/A_0_) ×100, where A_0_ represents the initial absorbance at time zero.

To explore the molecular basis underlying enhanced biofilm formation in the Δ*essC* mutant, the transcriptional levels of key regulatory genes associated with biofilm development were analyzed by quantitative real-time PCR. Under neutral conditions (CK, pH 7.2), no significant differences were observed in transcriptional levels of the regulatory genes between the wild-type and Δ*essC* deletion strains. Under acidic conditions, deletion of *essC* resulted in a modest downregulation of the quorum-sensing regulator *agrC* (Log_2_FoldChange_WT:Δ*essC*_ = 1.008 ± 0.156: 0.616 ± 0.039), whereas the expression levels of *arlS* (Log_2_FoldChange_WT:Δ*essC*_ = 1.011 ± 0.176: 2.823 ± 0.429; *P* < 0.05), *sigB* (Log_2_FoldChange_WT:Δ*essC*_ = 1.010 ± 0.169: 2.045 ± 0.089; *P* < 0.05) were significantly upregulated compared with the WT strain. Upon complementation of *essC*, the expression levels of *agrC*, *arlS*, and *sigB* were largely restored to wild-type levels (Fig. 2A), indicating that the transcriptional changes of these regulatory genes under acidic conditions depend on *essC* deletion.

Given that *arlS* showed the most pronounced change in the Δ*essC* mutant under acidic conditions, we further investigated the regulatory relationship between *arlRS* and *essC*. Using a fluorescence reporter system, we measured T7SS promoter activity in the USA300 Δ*arlRS* mutant at stationary phase under acidic (pH 5.0) and control (CK) conditions. In contrast to the wild-type strain, in which T7SS promoter activity was significantly upregulated under acidic conditions, deletion of *arlRS* abolished the acid-induced activation of T7SS transcription. Moreover, T7SS promoter activity in the Δ*arlRS* mutant was lower than that in the control group, suggesting a direct regulatory link between *arlRS* and the T7SS (Fig. 2B). The fluorescent reporter plasmid pCM29-P_T7SS_-GFP, which was previously constructed in our laboratory to monitor T7SS promoter activity.

In addition, *arlRS* is a positive regulator of *icaA*, a gene involved in PIA biosynthesis and biofilm formation. Upregulation of *arlRS* promotes *icaA* expression. Consistently, the transcriptional level of *icaA* was significantly upregulated in the USA300 Δ*essC* mutant cultured to stationary phase under acidic conditions (Log_2_FoldChange_WT:Δ*essC*_ = 1.005 ± 0.116: 2.28 ± 0.327; *P* < 0.05) (Fig. 2C). These results further validate the regulatory relationship and confirm that EssC deletion affects the “*arlRS*-*ica*” regulatory axis under acidic stress.

Consistent with increased *icaA* expression, CLSM analysis using wheat germ agglutinin (WGA) staining revealed markedly elevated levels of polysaccharide intercellular adhesin (PIA) in Δ*essC* mutant biofilms under acidic conditions. Firstly, to exclude nonspecific binding of WGA, we used the *icaA* transposon insertion mutant of *S. aureus* USA300 (USA300 Tn::*icaA*) and the *icaC* knockout mutant of *S. epidermidis* 1457 strain (1457Δ*icaC*) as negative control for PIA synthesis. Fluorescence quantification showed that, compared with the wild-type strain, the fluorescence intensity within the mutant biofilm was significantly reduced (Fig. 2D and E). These results validate the feasibility of using WGA staining to detect PIA synthesis. Therefore, we used the method described above to measure PIA content in biofilms. Consistent with the upregulation of *icaA* expression, CLSM imaging after WGA staining revealed a marked increase in PIA content in the Δ*essC* biofilm under acidic conditions. Quantitative fluorescence analysis further indicated an approximately 1.5-fold increase in PIA-associated signal intensity compared with WT biofilms (Fig. 2D and E). Given the central role of PIA in mediating intercellular adhesion, auto-aggregation assays were performed to assess bacterial aggregation capacity. The Δ*essC* mutant exhibited significantly higher auto-aggregation ratios at 6 h (Fig. 2F, *P* < 0.05), supporting the notion that enhanced PIA production promotes tighter cell–cell interactions during biofilm maturation.

### 3.4 Enhanced expression of fibronectin-binding proteins promotes initial attachment in the ΔessC mutant

**Fig. 3:**
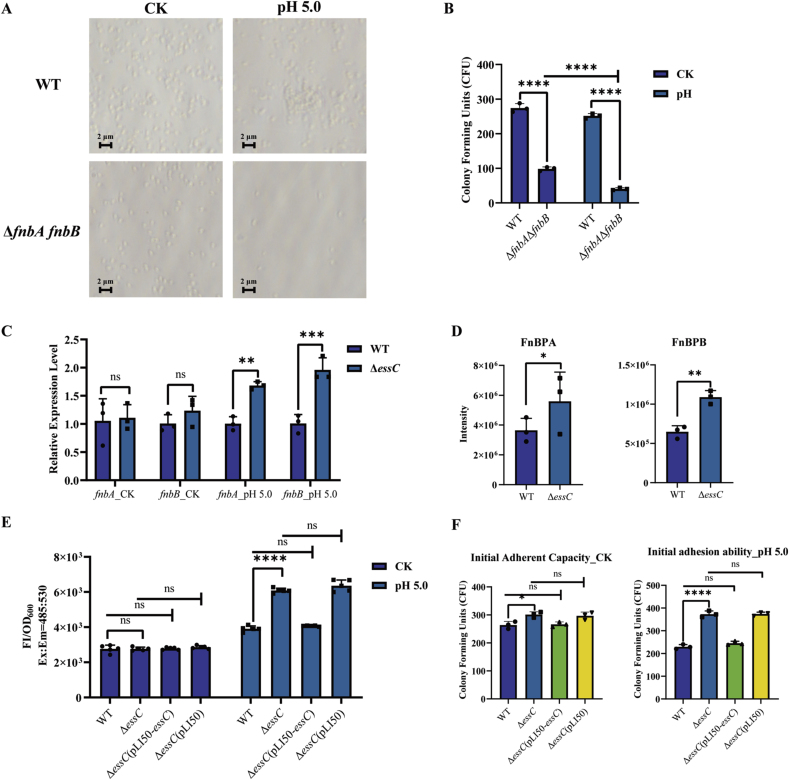
Enhanced expression of fibronectin-binding proteins promotes initial attachment in the Δ*essC* mutant (**A**) Representative images of the initial adhesion cells of *S. aureus* USA300 WT and Δ*fnbA fnbB* mutants attached to the surface of 6-well plates after 1 h of incubation under the control and acidic condition. **(B)** Quantitative analysis of initial adhesion cells number of *S. aureus* USA300 WT and Δ*fnbA fnbB* mutants to 6-well plates under the control and acidic condition. Statistical significance was determined by one-way ANOVA (n = 3; ∗∗∗∗: *P* < 0.0001). **(C)** Relative expression of *fnbA、fnbB* of *S. aureus* USA300 and Δ*essC* mutant strains at 4 h under the control and acidic condition. (**D**) Absolute expression intensity of FnbA、FnbB of *S. aureus* USA300 and Δ*essC* mutant strains at 16 h under the acidic condition. (**E**) Indirect immunofluorescence of FnBPA/B protein on the surface of *S. aureus* cells. (**F**) Quantitative analysis of initial adhesion cells number of *S. aureus* USA300 WT and *essC* mutants to 6-well plates under the control and acidic condition. Statistical significance was determined by one-way ANOVA (n = 3; ∗: *P* < 0.05; ∗∗∗∗: *P* < 0.0001; ns: No difference).

Because initial surface attachment is a critical early step in biofilm formation, the expression of fibronectin-binding protein genes *fnbA* and *fnbB* was examined. Firstly, to elucidate the direct contribution of *fnbA* and *fnbB* to bacterial initial adhesion, we further examined the attachment of the *S. aureus* NCTC 8325 wild-type strain and the Δ*fnbA fnbB* double-deletion mutant in 6-well plates. Microscopic imaging showed that deletion of *fnbA* and *fnbB* significantly impaired bacterial adhesion to the plates, and the acidic environment further reduced the number of adherent bacteria (Fig. 3A). These findings were confirmed by cell counting (Fig. 3B). Together, these results demonstrate a direct link between *fnbA*/*fnbB* and the initial adhesion capacity of *S. aureus*. Under acidic conditions, both genes were significantly upregulated in the Δ*essC* mutant at early (4 h) and later (16 h) time points, with *fnbB* showing nearly a two-fold increase in transcriptional level relative to the WT strain (Fig. 3C). Proteomic and immunofluorescence analyses further confirmed increased abundance of FnBPA and FnBPB proteins in the Δ*essC* mutant (Fig. 3D and E).

Functionally, enhanced expression of fibronectin-binding proteins correlated with increased initial attachment. Microscopic quantification revealed that more Δ*essC* mutant cells adhered to polyethylene surfaces within 1 h under acidic conditions compared with WT cells, with approximately a two-fold increase in attached bacteria per field (Fig. 3F). These data indicate that loss of EssC facilitates early-stage biofilm establishment by promoting adhesin-mediated surface attachment.

### 3.5 Increased autolysis and extracellular DNA accumulation stabilize ΔessC mutant biofilms

**Fig. 4:**
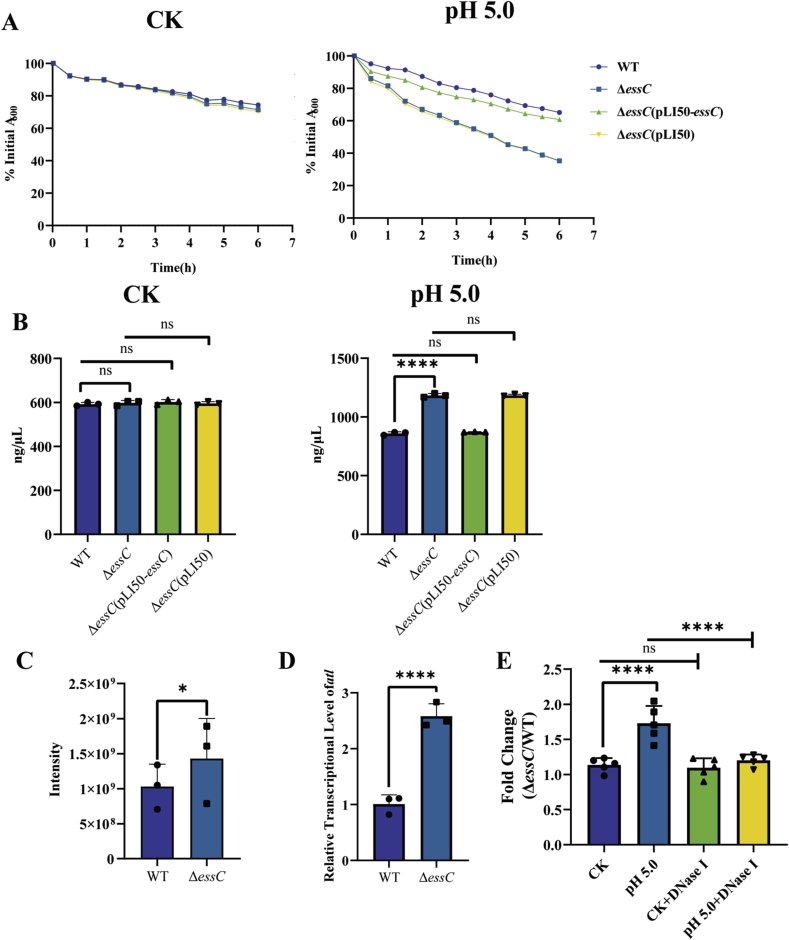
Increased autolysis and extracellular DNA accumulation stabilize Δ*essC* mutant biofilms (**A**) The autolytic activity of *S. aureus* USA300 WT and *essC* mutant strains under the control and acidic condition was determined by the Triton X-100-induced autolysis assay. The decrease in OD600 was monitored over 6 h. The autolysis rate was expressed as the percentage decrease in OD600 relative to the initial value (time zero). (**B**) Absolute expression intensity of Atl of *S. aureus* USA300 and Δ*essC* mutant at 16 h under the acidic condition. (**C**) Relative expression of *atl* under acidic condition of *S. aureus* USA300 and Δ*essC* mutant at 16 h compared to the control group detected by qRT-PCR. Statistical significance was determined by Unpaired *t*-test (n = 3; ∗∗∗: *P* < 0.0005). (**D**) Quatification of eDNA content in mature 48-h biofilms of *S. aureus* USA300 WT and *essC* mutant strains under acidic condition, extracted by a standard phenol-chloroform protocol and then measured by NanoDrop. Statistical significance was determined by one-way ANOVA (n = 3; ∗∗∗∗: *P* < 0.0001; ns: No difference). (**E**) Effect of DNase I treatment on biofilms formation of USA300 wild-type and *essC* mutant strains. Quantitative results of crystal violet staining. Fold change of biofilm formation = OD570_Δ*essC*/WT_. Statistical significance was determined by one-way ANOVA (n = 3; ∗∗∗∗: *P* < 0.0001; ns: No difference). (For interpretation of the references to colour in this figure legend, the reader is referred to the Web version of this article.)

Proteomic analysis identified the major autolysin Atl as one of the most strongly upregulated proteins in the Δ*essC* mutant under acidic conditions (Fig. 4C). This observation was corroborated by qPCR analysis, which revealed a greater than twofold increase in *atl* transcription (Fig. 4D). Given the role of Atl in cell wall turnover and autolysis, Triton X-100–induced autolysis assays were performed. The Δ*essC* mutant displayed accelerated autolysis compared with the WT strain, with a substantially greater reduction in OD600 over time under acidic conditions (Fig. 4A).

Enhanced autolysis was accompanied by increased accumulation of extracellular DNA (eDNA) within mature biofilms (Fig. 4B). Quantitative analysis demonstrated that eDNA concentrations were significantly higher in Δ*essC* mutant biofilms than in WT biofilms under acidic stress (eDNA _WT:ΔessC_ = 859.33 ng/μL: 1183.33 ng/μL, *P* < 0.05). Moreover, crystal violet staining clearly showed that after DNase I treatment, the biofilm biomass of all groups under both control (CK) and acidic (pH 5.0) conditions was significantly reduced compared to before treatment, consistent with the role of eDNA as an important extracellular matrix component in biofilm formation (Fig. 1A). We further used the ratio OD_570__Δ*essC*/WT_ as an indicator of the increase in biofilm formation. The ratio showed a significant difference before and after DNase I treatment. Specifically, after DNase I treatment, the magnitude of the increase in Δ*essC* biofilm formation was markedly smaller than before treatment, suggesting that eDNA plays an important role in the enhanced biofilm phenotype of Δ*essC* (Fig. 4E).

### 3.6 Altered cell surface properties contribute to enhanced bacterial aggregation

**Fig. 5:**
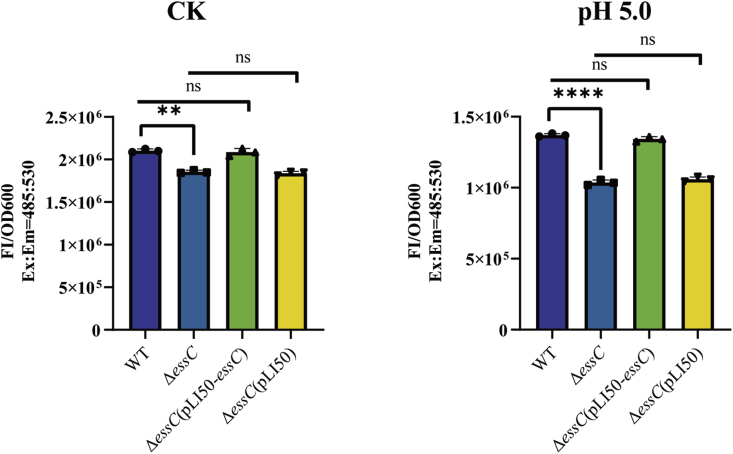
Altered cell surface properties contribute to enhanced bacterial aggregation Quantification of the surface charge of *S. aureus* USA300 WT and *essC* mutant strains with a fluorescein-labeled poly-L-lysine (PLL-FITC) probe. The bacteria were cultured in TSB (CK) and TSB (pH 5.0) at 37 °C overnight and then co-incubated with PLL-FITC for 30 min. Statistical significance was determined by one-way ANOVA (n = 3; ∗∗: *P* < 0.005; ∗∗∗∗: *P* < 0.0001; ns: No difference).

Finally, potential changes in bacterial cell surface properties were assessed using FITC-labeled poly-L-lysine binding assays. Under acidic conditions, the Δ*essC* mutant exhibited a modest but statistically significant reduction in surface negative charge compared with the WT strain (Fig. 5, FI/OD600 _Δ*essC*/WT_ = 0.75, *P* < 0.05). Reduced surface charge is expected to diminish electrostatic repulsion between bacterial cells, thereby facilitating intercellular aggregation. This physicochemical change likely acts in concert with increased PIA production and eDNA accumulation to promote robust biofilm maturation in the absence of EssC.

## 4 Discussion

Biofilm formation represents a central adaptive strategy that enables *Staphylococcus aureus* to persist under hostile environmental conditions [1,20]. In this study, we demonstrate that deletion of the T7SS ATPase EssC profoundly reshapes the biofilm phenotype of *S. aureus* under acidic stress. Although loss of EssC compromises bacterial viability, it simultaneously drives the formation of thicker and more structurally robust biofilms, highlighting a shift from planktonic growth toward a biofilm-oriented survival mode.

Previous studies on the *S. aureus* T7SS have primarily focused on its role in virulence, immune evasion, and interbacterial competition, largely through the secretion of effector proteins such as EsxA and EsxB [33,34,[39], [40], [41], [42], [43], [44]]. EssC has been established as an essential component of the secretion machinery, and its deletion results in a nonfunctional T7SS [33,38,[45], [46], [47]]. Our findings extend the functional scope of EssC beyond secretion and virulence, revealing its involvement in environmental adaptation and biofilm regulation under acidic conditions. This observation aligns with emerging evidence that secretion systems can exert broad regulatory effects on bacterial physiology beyond their canonical transport functions [48,49].

One of the most striking features of the Δ*essC* mutant was the coordinated transcriptional remodeling of global regulatory networks associated with biofilm formation, and this change depends on a low pH environment. The close linkage among “environmental signal – gene expression – phenotypic change” suggests that *essC*/T7SS may represent a previously unrecognized but important node in the acid stress response network of *S. aureus*. Elucidating this mechanism will not only deepen our understanding of the survival strategies of *S. aureus* but also provide a theoretical basis for developing novel antibacterial strategies that target its environmental adaptation mechanisms.

Compared with *M. tuberculosis*, studies on the environmental signals that trigger the T7SS in *S. aureus* remain relatively limited. In *M. tuberculosis*, the T7SS is subject to complex regulation. For example, the expression of ESX-1 is transcriptionally regulated by the PhoPR two-component system [50], which responds to low-pH signals and is closely associated with phagosomal colonization [51]. ESX-5 expression is induced by phosphate starvation [52], whereas ESX-3 expression is significantly suppressed under iron or zinc limitation [53]. Regarding environmental factors, Chatterjee et al. demonstrated that bacteriophage infection or exposure to sub-inhibitory concentrations of antibiotics (e.g., daptomycin, ciprofloxacin) activates T7SS expression in enterococci, which is considered an adaptive stress response [54]. In contrast, the activating factors of the *S. aureus* T7SS remain poorly understood, and most known factors are confined to the host immune microenvironment. For instance, Ishii et al. reported that lung surfactant induces high expression of T7SS-related genes in *S. aureus*, thereby enhancing bacterial survival and immune evasion in the mouse lung [55]. Casabona et al., using transcriptomic and metabolomic analyses, demonstrated that hemoglobin and its cofactor heme B activate the T7SS of *S. aureus* strain RN6390 at both the transcriptional and post-translational levels, revealing the important role of this system in iron homeostasis [56].

Although it has been reported that transcription of the *S. aureus* T7SS is regulated by a complex network, direct evidence remains scarce. Specifically, SigB modulates multiple downstream factors either directly or indirectly. Among these, the DNA-binding protein SpoVG is directly positively regulated by SigB and activates the T7SS, whereas SarA, another direct target of SigB, strongly represses the T7SS [57]. The two-component system ArlRS also positively regulates the T7SS, but its transcription is indirectly influenced by SigB [33]. In addition, the quorum-sensing system Agr positively regulates the T7SS, yet the expression of its effector molecule RNAIII is suppressed by SigB [29]. Outside the SigB network, the two-component system SaeRS is an important negative regulator of the T7SS [33]. For example, in the *S. aureus* Newman strain, sustained activation of the SaeS kinase results in low T7SS activity [58]. Nevertheless, direct experimental evidence supporting the regulatory relationship between these factors and *essC*/T7SS is still lacking.

In this study, downregulation of the *agr* quorum-sensing system is well known to favor biofilm accumulation by relieving repression of surface adhesins and the *icaADBC* operon [20,59]. Consistent with this paradigm, reduced *agrC* expression in the Δ*essC* mutant was accompanied by increased expression of *icaA* and enhanced PIA production. In parallel, upregulation of the *arlS-icaA* and *sigB-icaA* axis further reinforced PIA synthesis and biofilm maturation [60,61]. Similar regulatory configurations have been reported in response to other environmental stresses, suggesting that EssC may influence biofilm formation by modulating stress-responsive signaling pathways. Moreover, we employed a fluorescence reporter system to preliminarily validate the regulatory role of ArlRS on T7SS genes under acidic conditions. This provides a new approach for understanding the regulatory mechanism of the T7SS. Our findings not only expand the spectrum of activating signals for the *S. aureus* T7SS but also offer a basis for further elucidating the adaptive strategy of this pathogen in acidic niches.

Enhanced initial attachment in the Δ*essC* mutant was mediated, at least in part, by increased expression of fibronectin-binding proteins FnBPA and FnBPB. These MSCRAMMs play critical roles during the early stages of biofilm development by promoting adhesion to host tissues and abiotic surfaces [62,63]. Previous studies have shown that acidic pH can modulate adhesin expression [20,[64], [65], [66], [67]]; our data indicate that EssC acts as an additional regulatory layer that constrains adhesin-mediated attachment under acidic stress. By releasing this constraint, *essC* deletion allows rapid surface colonization, potentially conferring a competitive advantage in acidic niches.

Another key finding of this study is the pronounced increase in autolysis and eDNA release observed in the Δ*essC* mutant. The major autolysin Atl of *S. aureus* not only participates in bacterial autolysis and normal daughter cell separation but also plays key roles as a multifunctional virulence factor. It promotes biofilm formation by releasing eDNA through lysis; acts as an adhesin mediating bacterial binding to host extracellular matrix proteins such as fibronectin [[17], [18], [19],^68^]; facilitates invasion into non-professional phagocytes via interaction with the host Hsc70 receptor to achieve immune evasion [69]; and is involved in the non-classical secretion of cytoplasmic proteins [70]. Furthermore, Atl can activate the host innate immune response by releasing peptidoglycan fragments [71]. While in other words, the increased eDNA content within the Δ*essC* biofilm under acidic conditions involves multiple factors, including but not limited to: EssC deficiency reduces the resistance of *S. aureus* to the acidic environment, leading to increased bacterial death and consequently more eDNA release; the elevated expression of the autolysin Atl enhances autolysis, accompanied by additional eDNA release and accumulation. The synergistic effect of these factors promotes eDNA accumulation, thereby shaping a more robust biofilm structure in the Δ*essC* strain under acidic conditions.

In addition to regulatory and matrix-related factors, changes in bacterial cell surface properties also contributed to the hyper-biofilm phenotype [72,73]. Potential reasons for the reduction in bacterial cell wall surface negative charge upon EssC deletion under acidic conditions: likely the synergistic effects of acidic pH, cell membrane damage, and resource allocation strategies under stress. First, the charge state of chemical groups on the bacterial surface, such as the phosphate groups of teichoic acids, is directly determined by the environmental pH. Under acidic conditions, these groups bind more protons (H^+^), leading to a reduction in negative charge [74]. Second, based on the SYTO-9/PI staining results showing exacerbated cell membrane damage within the Δ*essC* biofilm under acidic conditions, we hypothesize that changes in membrane lipid composition indirectly affect cell wall charge [75]. EssC deficiency may interfere with the adaptive regulation of the bacterium to the acidic environment, resulting in abnormal upregulation of LPG or other membrane lipids and consequently reducing the net surface negative charge. Third, acid stress forces the bacterium to allocate limited resources toward repair and resistance rather than building an intact cell wall, which may further indirectly reduce the synthesis efficiency of components such as teichoic acids and exacerbate cell wall defects. This is consistent with previous reports showing that T7SS mutants (including strains lacking EssC, EsxA, or EsxC) exhibit altered cell surface morphology and abnormal cell wall synthesis under normal growth conditions [76]. The modest reduction in surface negative charge observed in the Δ*essC* mutant is expected to weaken electrostatic repulsion between cells, thereby facilitating aggregation. Although this effect alone is unlikely to fully account for the enhanced biofilm formation, it likely acts synergistically with increased PIA and eDNA production to promote biofilm maturation.

**Fig. 6:**
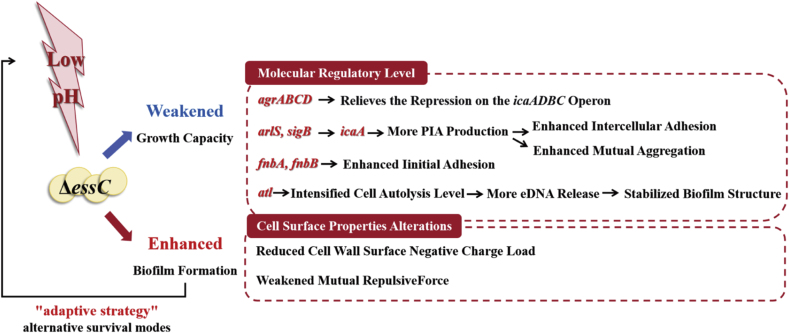
Loss of EssC triggers a comprehensive adaptive remodeling of *S. aureus* physiology under acidic stress *essC* deletion reprograms multiple stages of biofilm development, including enhanced initial adhesion mediated by upregulated fibronectin-binding proteins (FnBPA and FnBPB), increased intercellular aggregation driven by elevated polysaccharide intercellular adhesin (PIA) production, and biofilm stabilization through augmented autolysis-dependent extracellular DNA release. These phenotypic changes are accompanied by coordinated transcriptional remodeling, characterized by downregulation of the biofilm repressor *agr* and activation of the *arlS–icaA and sigB–icaA* regulatory axis.

Taken together, our results support a model in which loss of EssC triggers a comprehensive adaptive remodeling of *S. aureus* physiology under acidic stress. Rather than maximizing growth, the bacterium adopts a defensive strategy characterized by reduced viability but enhanced community-level protection through biofilm formation. This trade-off between individual fitness and collective resilience may represent a general principle governing bacterial survival in hostile environments (Fig. 6).

In conclusion, this study identifies EssC as a previously unrecognized modulator of biofilm formation in *S. aureus* under acidic stress. By linking the T7SS core machinery to global regulatory networks, adhesin expression, autolysis, and extracellular matrix production, our findings provide new insights into the multifaceted roles of secretion systems in bacterial adaptation. These insights may inform future strategies aimed at disrupting biofilm-associated persistence and improving the treatment of chronic *S. aureus* infections.

## CRediT authorship contribution statement

**Yang Zhang:** Data curation, Resources, Writing – original draft. **Zihui Wang:** Methodology, Resources. **Lan Wei:** Methodology, Resources. **Deyu Wang:** Resources. **Di Qu:** Conceptualization. **Youhua Xie:** Conceptualization. **Yang Wu:** Conceptualization, Funding acquisition, Writing – review & editing.

## Declaration of competing interest

The authors declare the following financial interests/personal relationships which may be considered as potential competing interests: Yang Wu reports financial support was provided by National Natural Science Foundation of China. If there are other authors, they declare that they have no known competing financial interests or personal relationships that could have appeared to influence the work reported in this paper.

## Data Availability

Data will be made available on request.
